# Liposomes, transfersomes and niosomes: production methods and their applications in the vaccinal field

**DOI:** 10.1186/s12967-024-05160-4

**Published:** 2024-04-09

**Authors:** Domenico Riccardi, Lucia Baldino, Ernesto Reverchon

**Affiliations:** Department of Industrial Engineering, University of Salerno, Via Giovanni Paolo II, 132, 84084 Fisciano, SA Italy

**Keywords:** Liposomes, Transfersomes, Niosomes, Vaccines, Immune response, Antibodies, Supercritical carbon dioxide

## Abstract

One of the most effective strategies to fight viruses and handle health diseases is vaccination. Recent studies and current applications are moving on antigen, DNA and RNA-based vaccines to overcome the limitations related to the conventional vaccination strategies, such as low safety, necessity of multiple injection, and side effects. However, due to the instability of pristine antigen, RNA and DNA molecules, the use of nanocarriers is required. Among the different nanocarriers proposed for vaccinal applications, three types of nanovesicles were selected and analysed in this review: liposomes, transfersomes and niosomes. PubMed, Scopus and Google Scholar databases were used for searching recent papers on the most frequently used conventional and innovative methods of production of these nanovesicles. Weaknesses and limitations of conventional methods (i.e., multiple post-processing, solvent residue, batch-mode processes) can be overcome using innovative methods, in particular, the ones assisted by supercritical carbon dioxide. SuperSomes process emerged as a promising production technique of solvent-free nanovesicles, since it can be easily scaled-up, works in continuous-mode, and does not require further post-processing steps to obtain the desired products. As a result of the literature analysis, supercritical carbon dioxide assisted methods attracted a lot of interest for nanovesicles production in the vaccinal field. However, despite their numerous advantages, supercritical processes require further studies for the production of liposomes, transfersomes and niosomes with the aim of reaching well-defined technologies suitable for industrial applications and mass production of vaccines.

## Introduction

Vaccination is one of the most effective and cheap strategies to handle health diseases and fight viruses. Different studies state that vaccines prevent every year more than 2.5 million deaths worldwide [1]. Recently, the vaccination strategy has been widely used to avoid hospitalization, severe symptoms and death caused by the SARS-CoV-2 virus, showing very high effectiveness and giving the possibility to overcome a world pandemic [2].

Vaccines are now sparking more interest, and new types and strategies of vaccines are going to be developed and used for different medical issues, such as autoimmune disorders and certain types of cancer [3].

De Gregorio et al. [1] reported that the first witness of immunity from a disease can be found in Thucydides’ “The History of the Peloponnesian War”, in which the plague that was affecting Athens is described, stating that “the disease was not able to infect twice the same subject”. Until 1796, the only way to spread immunity, in particular for the smallpox disease, was the variolation, which consisted of inoculating materials taken from an infected patient, inducing a mild form of the disease and, thus, preventing the person from being re-infected. This technique was not safe because the risk of contracting the disease in a fatal way was possible [4]. Edward Jenner, in 1796, used pustules from cowpox to induce smallpox immunity, having the same results of variolation; but with much less severe side effects [5]. This event is set as the birth of the first vaccine, opening the way to a new approach for their development. Subsequently, vaccines were based on weakening and attenuating viruses before inoculating them into the patient using oxygen or heat, as first performed by Louis Pasteur in 1880 for chicken cholera and anthrax [6, 7]. Vaccines for diphtheria and tetanus were developed in 1923, when Alexander Glenny and Barbara Hopkins used formaldehyde to inactivate bacterial toxins [8]. Polysaccharides vaccines were introduced in 1960 for meningococcal prevention [9].

However, the introduction of new technologies became significantly important for the development of safer and more effective vaccines. Indeed, though the first phase of research on vaccine was useful for the development of conventional vaccines formed by inactivated microbes that were able to induce an immune response, multiple injections were needed and they tended to have a poor safety record [10]. To overcome these disadvantages, novel formulations of vaccine have been developed. Subunit vaccines do not contain the whole pathogen; but only the antigenic part of the pathogens, such as proteins, polysaccharides or peptides. Moreover, these types of vaccines present numerous advantages, such as application to immunocompromised patients and less chances of causing side effects [11]. On the other hand, they present limitations like reduced immunogenic action than the conventional vaccines and the necessity to add adjuvants to the formulation, that are substances that can enhance the immune response [12, 13]. One of the ways to overcome this problem is to encapsulate antigens in nanometric particles that can also protect them from degradation [14].

The necessity of using nanometric particles as nanocarriers is also found for DNA and RNA-based vaccines due to their fast degradation when used in their ‘naked’ form. For this reason, advanced delivery vesicles, like liposomes, transfersomes and niosomes, have been proposed and used [15], to protect these macromolecules from degradation before and after cell transfections [16, 17].

Nanovesicles, like liposomes, transfersomes and niosomes show an adjuvant ability, enhancing the immune response to antigens encapsulated in their aqueous core [14, 18]. RNA-based vaccines, especially mRNA-based vaccines, have been recently developed and approved by the FDA to fight the COVID-19 pandemic [19]. mRNA in a cell can be translated by ribosomes in the cytoplasm forming the required protein (this process is called protein synthesis). In particular, for vaccine utilization, mRNA is synthesized to transport the information of only the antigen production; i.e., mRNA is recognised by ribosomes, the message is decrypted, and antigens are produced [20]. Antigens are exposed on the surface of APC cells (antigen-presenting cells, like dendritic cells) and, consequently, lymphocytes T cells, called helpers, induce the immunity response recognising the antigen as a threat to the organism, stimulating the formation of cytotoxic T lymphocytes that are able to kill the infected cells, and the formation of B lymphocytes cells that produce specific antibodies; B lymphocytes can evolve in memory B cells, which are able to preserve the capacity of recognise the virus, even after years from the first infection/vaccination, in order to re-activate in a fast and more efficiently way the specific immune response in case of infection [21].

**Fig. 1:**
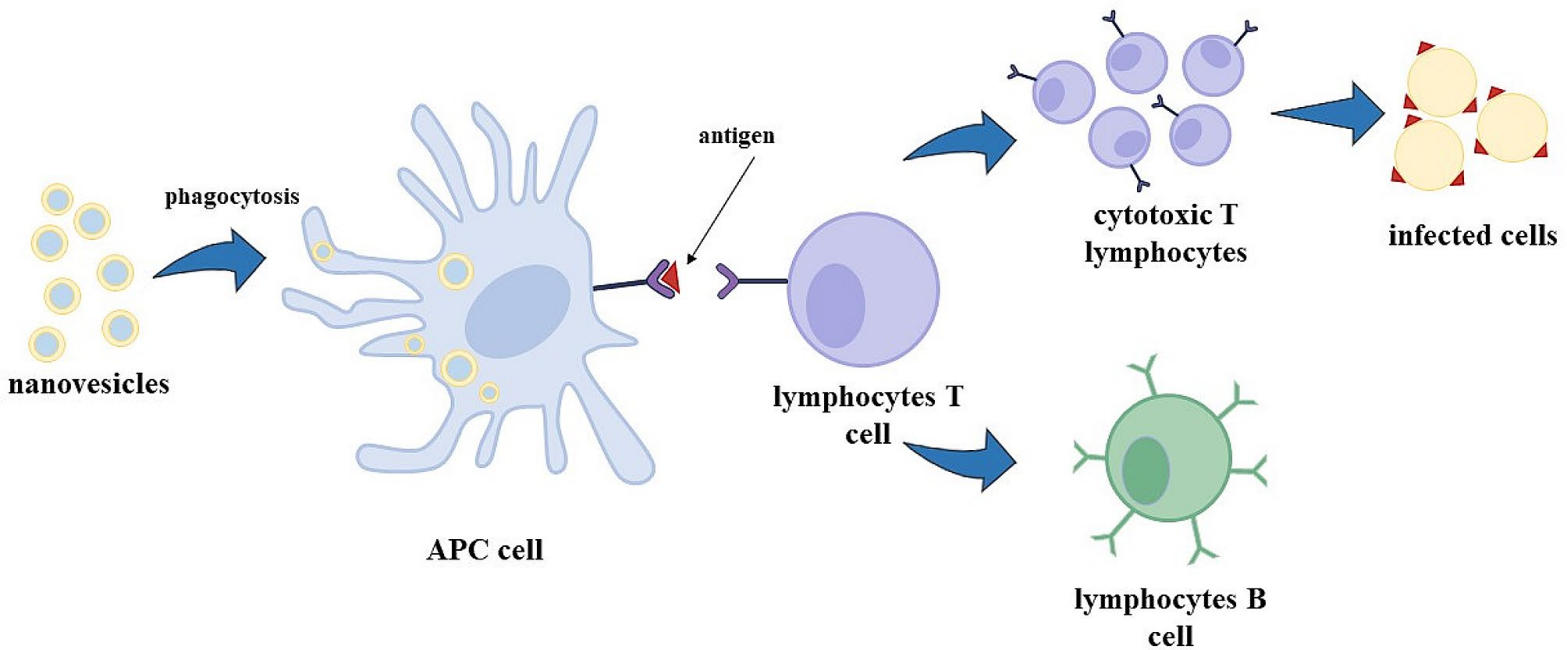
Mechanism of immune response

Lipid-based nanovesicles are internalized by APCs throughout phagocytosis (Fig. 1) or receptor-mediated endocytosis: in particular, carriers smaller than 150 nm are internalized by endocytosis, whereas vesicles bigger than 150 nm are taken up by APCs through phagocytosis [22].

Therefore, this review is focused on the application of these nanovesicles in the vaccinal field, and the advantages/disadvantages of their main production techniques are critically discussed in view of the requirements of the industrial scale-up.

## Typesof nanovesicles

**Fig. 2:**
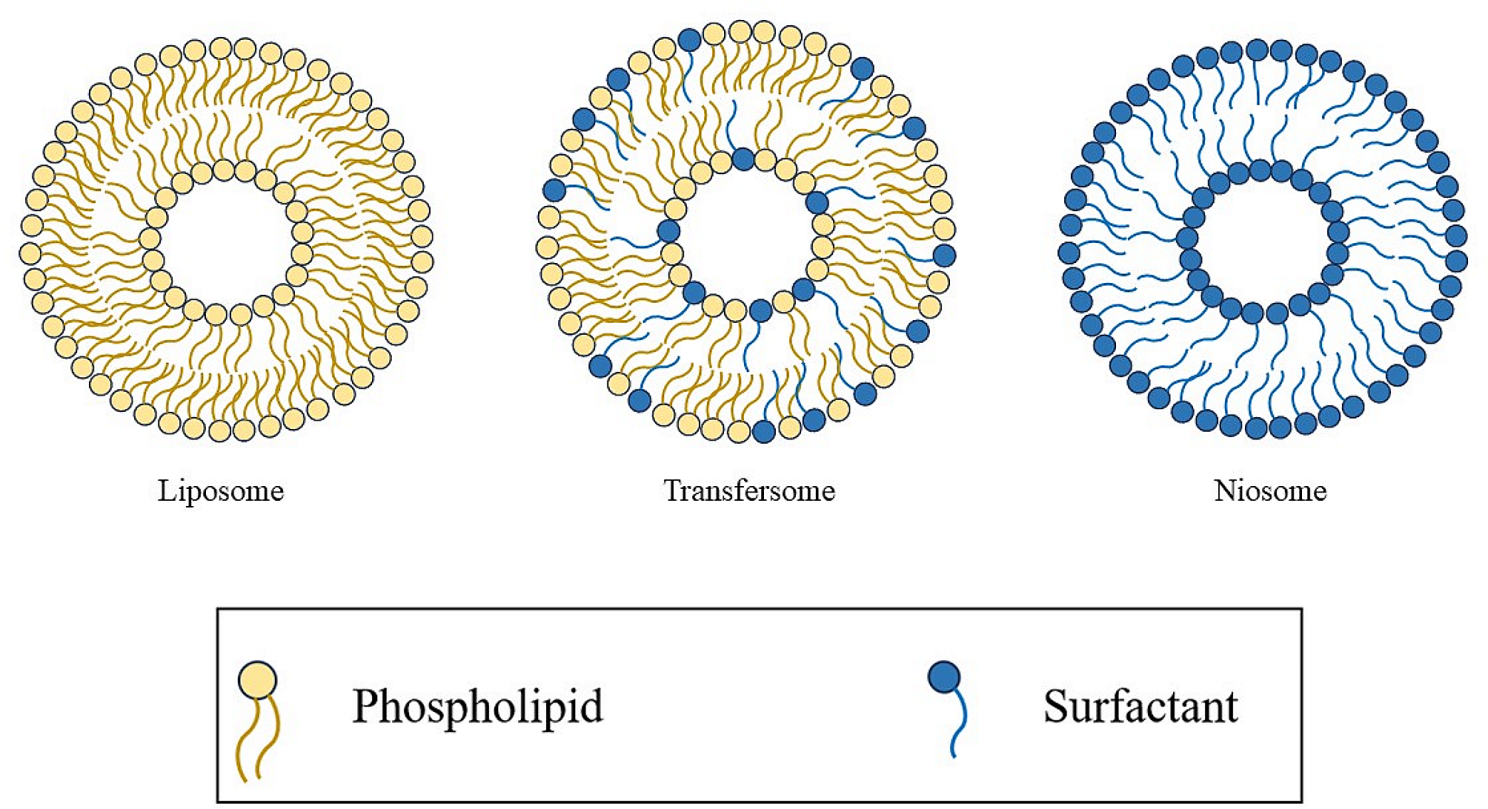
Liposome, transfersome and niosome structure

Liposomes are vesicles characterized by an aqueous core surrounded by a double layer of phospholipids that are amphiphilic compounds with a hydrophilic head and hydrophobic tails [23]. Due to the amphipathic nature of the phospholipids, they can spontaneously rearrange their structure into a spherical geometry, in an aqueous environment (Fig. 2); moreover, they are able to carry both hydrophilic compounds in the inner core, and lipophilic compounds in the external double-layer [24]. Owing to this property, they are used, among the others, to deliver antioxidants, anti-inflammatory, antimicrobial and anticancer compounds [25].

Liposomes structure can also be modified to obtain transfersomes: they are known as ultra-deformable liposomes because of their flexible membrane that allows them to deform and penetrate the skin in an effective way [26]. Transfersomes can be obtained by adding to the lipidic layer compounds called penetration enhancers or edge activators that are surfactants, such as sodium deoxycholate, span and tween [27] (Fig. 2). These edge activators produce a discontinuity in the nanovesicle membrane allowing the deformation of the transfersomes. The advantage of using transfersomes is the easierway of drug administration throughout the skin; they have indeed the ability to permeate the skin and can encapsulate different types of compounds like small molecules, proteins, antioxidants, peptides and vaccines [28].

Niosomes are nanovesicles formed by non-ionic surfactants. They are similar to liposomes in terms of physical properties (Fig. 2); but, they show a longer physical-chemical stability (even at room conditions), lower toxicity because of their non-ionic nature, are biodegradable and non-immunogenic [29]. They have been used to encapsulate various compounds, such as chemotherapeutic drugs, antibiotics, antioxidants and vaccines [30, 31].

According to the kind of phospholipids used, liposomes, transfersomes and niosomes can show a negatively or positively surface charge; generally cationic liposomes are preferred for vaccine delivery, because APCs are negatively charged, and the electrostatic interactions can lead to a better cellular adhesion of nanovesicles to APCs, resulting in an accelerated phagocytosis [32–34]. For this purpose, different cationic phospholipids are used such as N-[1-(2,3-dioleoyloxy) propyl]-N, N,N-triethylammonium chloride (DOTMA), 1,2-dioleoyl-sn-glycerol-3-phosphoethanolamine (DOPE), N1, N1-dimyristeroyloxyethyl-spermine, 1,2-dioleoyl-3-trimethylammonium-propane (DOTAP) and others that will be mentioned in the subsequent sections.

The geometry of liposomes, transfersomes and niosomes depends on the critical packing parameter (CPP), defined in Eq. 1, where *V* is the volume of the non-polar group, *l*_*C*_ is the critical length of the non-polar group, and *a*_*0*_ is the area of the head polar group.

1 $$ CPP=\frac{V}{{l}_{C}\cdot {a}_{0}} $$

**Fig. 3:**
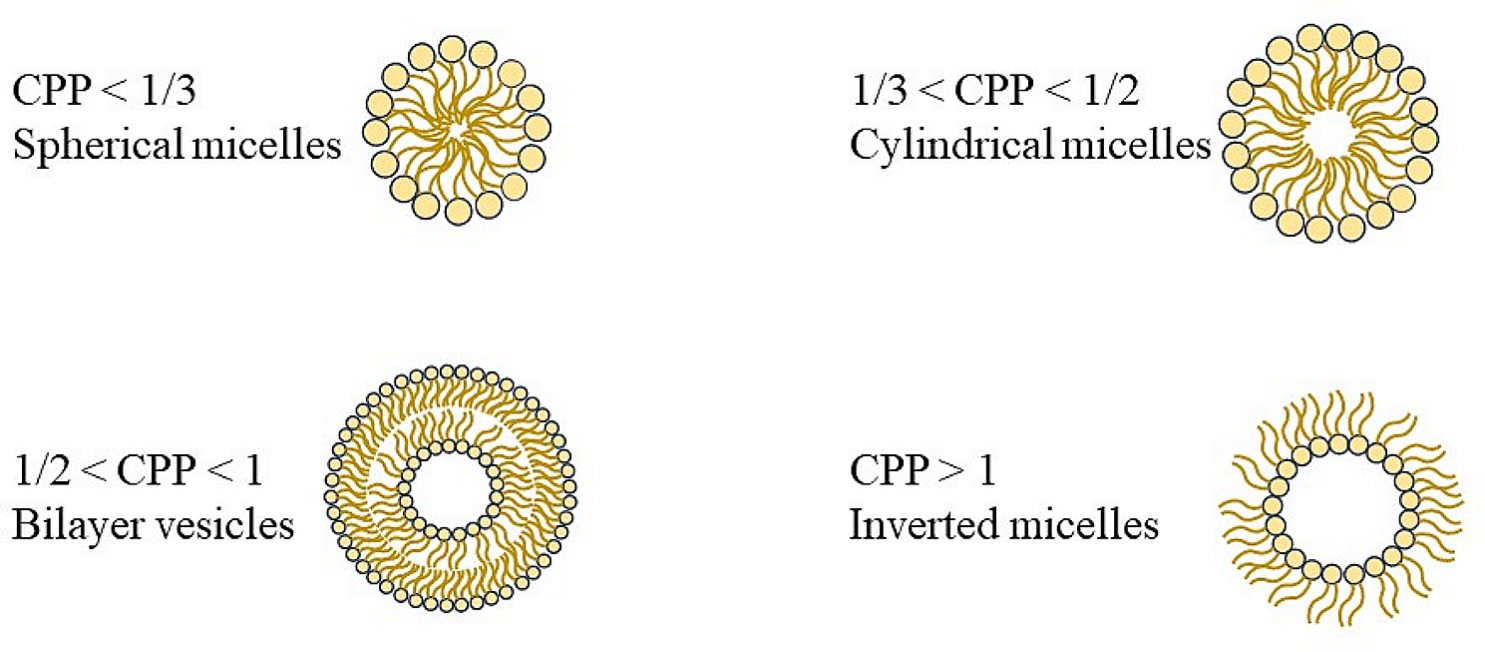
Arranged geometry of phospholipids based on CPP value

According to the CPP value, the following re-arranged geometry of phospholipids/surfactant can be obtained: spherical micelles for CPP ≤ 1/3, cylindrical micelles for 1/3 ≤ CPP ≤ 1/2, bilayers for 1/2 ≤ CPP ≤ 1 and inverse micelles for CPP > 1 (Fig. 3) [35, 36].

## Production of nanovesicles

Different methods were proposed to produce liposomes, transfersomes and niosomes. They can be divided in conventional or traditional methods (thin film hydration method, spray-drying method, injection method, reverse phase evaporation, etc.) and innovative methods (microfluidic method, supercritical methods). In this review, the most frequently used conventional and innovative methods of production of these nanovesicles are discussed.

### Conventional methods

#### Thin film hydration method

Thin film hydration method, known as the Bangham method, has been the first production technique of liposomes. In this method, phospholipids are dissolved in an organic solvent, such as ethanol, chloroform or methanol, and the solvent is then evaporated forming a thin lipid layer film on the flask wall. Subsequently, the lipids are hydrated using an aqueous or phosphate buffer solution containing the hydrophilic drug, with the consequent formation of a vesicle suspension. This production method shows different disadvantages: the solvent removal is a time consuming step, and it can take several hours of exposure to vacuum; moreover, this method does not allow a good control on vesicles size and size distribution. For this reason, a sonication step is required to obtain nanovesicles instead of microvesicles. Furthermore, the encapsulation efficiency is generally low, and the process works in a batch-mode [37–39].

#### Spray-drying method

In this method, phospholipids are dissolved in an organic solvent or in a mixture of organic solvents, such as methanol and chloroform, and mixed using a magnetic stirrer; the encapsulating compound is added to the mixture, first sonicated and then it is spray-dried at different conditions of temperature (even at 120 °C) [40, 41]. The obtained dried powder is hydrated using water or a phosphate buffer solution. Using this method, large vesicles are generally obtained (ranging from 300 to 600 nm) [42, 43]; therefore, membrane extrusion and/or sonication are used after their production [41, 42].

#### Injection method

In this method, an ethanolic solution containing phospholipids is prepared and, then, it is injected into a large volume of water or phosphate buffer; in this way, phospholipids tend to arrange in a bi-layer fragment. After the precipitation of these fragments in the aqueous phase, they tend to fuse to achieve a minimal length to finally form a close system and both, large and small unilamellar vesicles, can be obtained. The main disadvantages related to this method are the removal of residual ethanol, especially after azeotrope formation, low encapsulation efficiency and the obtained liposomal suspension is generally diluted [44]. Indeed, the volume of ethanol represents a key parameter to the formation of nanovesicles: if it is not higher than 7.5% v/v of the final volume of suspension and it is injected slowly, large and small unilamellar vesicles are formed. If the injection step is fast, multilamellar vesicles are formed [45]. Another method of injection uses ether: in this case, the problem of solvent removal can be overcome because ether and water are not miscible and can be easily removed by rotary evaporation. Encapsulation efficiency is higher than the one obtained in the ethanol injection method; but, vesicle morphology is not well controlled and mainly large unilamellar vesicles are formed [46]. Moreover, active compounds during this process are exposed to organic solvents at high temperature (ranging from 55 to 65 °C), and this can compromise the safety and the stability of the formulation [47].

#### Reverse phase evaporation

The lipid mixture, formed by phospholipids and an organic solvent, such as ethanol, diethyl ether or isopropyl ether, is inserted in a round flask and the solvent is evaporated under reduced pressure using a rotary evaporator; the lipids are then re-dissolved in the organic phase to form inverted micelles and the aqueous solution containing the drug is added, while the system is sonicated and kept under a nitrogen environment. The obtained mixture is evaporated to remove the residual organic solvent and the system becomes first a gel structure and, then, the structure collapses forming the suspension. In this method, a large amount of solvent is used; therefore, the problem of residual solvent in the suspension is particularly relevant [48].

### Innovative methods

Despite the batch conventional methods mentioned before are very easy to perform and do not require specific and elaborated equipments, they all present several limitations and further downstream steps of homogenization of the products are required [49]. For these reasons, innovative methods of production were investigated over the years to meet industrial requirements, such as the microfluidic method and methods assisted by supercritical CO_2_.

#### Microfluidic method

This method is similar to the traditional injection method; but a controlled mixing between the lipid phase and the aqueous phase is possible using a microfluidic channel having a 300 μm dimension width and 130–150– 200 μm dimension height [50–52], with different geometries [36]. Drug encapsulation efficiency can be over 70% [52]; but, disadvantages, such as the complexity of the microfluidic design, probability of channel clogging and dilute sample concentrations, can occur [49, 53].

#### Supercritical methods

More innovative methods are based on the utilization of CO_2_ at supercritical conditions (SC-CO_2_). Different processes were proposed during the year such as depressurization of an expanded solution into an aqueous medium (DESAM) that consists of the solubilization of lipids in an organic solvent, pressurization of the solution by adding CO_2_ in a dense state to obtain an expanded liquid and lastly the injection of the expanded liquid in an aqueous media. The gas and the solvent can be separated and recycled; the droplets formed during the depressurization step help in the formation of homogeneous liposomes and the dense gas is also used as agitation to improve size and homogeneity of the vesicles produced [54]. This process can produce liposomes characterized by diameters ranging from about 120 nm to 387 nm; however, in some cases, multimodal distributions are obtained.

Rapid expansion of a supercritical solution (RESS) consists of a two steps preparation: a solution containing lipids is dissolved in an organic solvent and put in contact with SC-CO_2_ in a vessel; subsequently, a depressurization step throughout a nozzle favours the formation of a lipid layer around the droplets, with consequent formation of liposomes [55].

Another process assisted by SC-CO_2_ is the supercritical reverse phase evaporation (SCRPE) [56]. A solution formed by phospholipids and ethanol is loaded in a variable volume cell and carbon dioxide is introduced in it. Then, pressure is reduced to completely release CO_2_, obtaining a liposomal dispersion [57].

A recently proposed supercritical process is SuperSomes. In this case, an ethanolic solution containing phospholipids, and eventually a hydrophobic drug, is fed to a saturator that is a high-pressure static mixer, using a high-precision pressure pump; CO_2_ is also pumped to the saturator. High-pressure mixing in the saturator produces a gas expanded liquid that is fed to the chamber throughout an injector. The aqueous phase, containing the hydrophilic drug, is fed to this chamber using a micrometric injector to produce small water droplets. In the chamber, inverted micelles are formed, since the hydrophilic heads of phospholipids arrange themselves around the sub-micron water droplets; whereas, on the bottom of the chamber, the presence of a water bulk allows the re-arrangement of phospholipids around the inverted micelles. The liposomal suspension is accumulated into a reservoir located downstream of the chamber and, opening at fixed time intervals an on-off valve, the suspension can be collected. The process can work in a continuous-mode by adding the ethanolic and aqueous solution and withdrawing the vesicle suspension. Moreover, throughout a depressurization line, the organic solvent can be completely removed and recovered in a separator vessel because of the solubility of ethanol in SC-CO_2_ [58, 59]. This one-shot process allows the production of large volumes of vesicle suspension and can be used to form liposomes [58, 59], niosomes [60, 61] and transfersomes [62] characterized by high drug encapsulation efficiency, even more than 90%, good control on vesicle size, size distribution and morphology, and does not require further downstream steps of particle reduction or purification [63].

## Nanovesicles-based vaccines

### Liposomes-based vaccines

Different formulations of liposomes are in trials for therapeutic vaccines against malaria, influenza, tuberculosis, and HIV. Other formulations are already commercially available against infection by human papillomavirus (HPV), influenza virus, hepatitis A virus [64], and COVID-19 [65].

One of the characteristics influencing the adjuvant efficacy of liposomes is the superficial charge: cationic liposomes exhibit a major interaction with the mucosal surfaces, prolonging the exposure time of the antigen, and show the ability to form complexes together with biomacromolecules, promoting the delivery of antigens to APCs [66]. For this reason, numerous studies and formulations using cationic liposomes have been proposed.

Heuts et al. [67] prepared cationic liposomes for antigenic peptides delivery using DOTAP (1,2-dioleoyl-3-trimethylammonium-propane) and DOPC (1,2-dioleoyl-sn-glycero-3-phosphocholine) lipids by the thin film hydration method. They found that cationic liposomes were suitable for encapsulating peptides used in therapeutic cancer vaccines, increasing the immune response. In particular, 15 peptides were encapsulated in different formulations, obtaining diameters from 125 nm up to 180 nm, values of Zeta potential from + 20 mV to + 35 mV and an encapsulation efficiency up to 79%. In vitro tests were performed to determine the immunological properties of the vesicles; five formulations showed levels of T cells activation similar to the free peptides introduced in the cell line, whereas the remaining 10 formulations showed a better action than free peptides.

Surface modification of liposomes, such as the insertion of hydrophilic polymers, like polyethylene glycol (PEG), chitosan and others, is a practice used to produce the so called “stealth” liposomes that cannot be detected by macrophages and opsonized, to better deliver the encapsulated compounds. Zhuang et al. [68] produced DOTAP PEGylated (DSPE-PEG_2000_) liposomes using the thin film hydration method, encapsulating ovalbumin (OVA), a key reference protein for vaccination experimentations. They demonstrated that the addition of 1 mol% DSPE-PEG_2000_ was able to enhance the uptake of APCs up to almost 40% with respect to non-pegylated liposomes; whereas, the addition of 5 mol% PEG produced a decrease of the uptake percentage (about 5%). Moreover, they demonstrated that PEGylation enhanced passive lymph nodes (where APCs were present) targeting of liposomes. Shimizu et al. [69] produced a system encapsulating antigen (Ag) to splenic marginal zone via PEGylated liposomes. The formulation induced an efficient immune response; however, several treatments of immunizations were required to achieve a strong antitumor effect. For this reason, the liposomal structure was modified by adding an adjuvant (α-galactosylceramide, a synthetic glycolipid with a strong immunostimulant action [70]), obtaining a strong response from the immune system. Furthermore, the formulation showed a prevention action and an efficient suppression of tumour growth. Yuba et al. [71] modified the superficial charge and structure of liposomes to obtain a larger immune response; they added a cation lipid to obtain adjuvant properties because of the consequent increase of the cellular association of liposomes to dendritic cells. In addition, cationic lipids helped to modify the superficial structure with β-glucan-based pH-responsive polysaccharides that contained anionic carboxy groups; they were added to the formulation because of their adjuvant properties of promoting cytokines production that are polypeptide mediators and play an important role in the communication among immune system cells. Moreover, they show cytodestructive effects on cancer cells or infectious agents [72, 73].

Summarizing, PEGylation of liposomes can enhance, as previously described, the residence time in the blood circulation of the nanovesicles; PEG can reduce protein absorption of nanovesicles by proteins like opsonins and lipoproteins. Despite these advantages, Schmidt et al. [74] reported a reduced binding and uptake phenomenon of liposomes by APCs, causing a loss of vaccine; in particular, the uptake level of PEGylated liposomes was reduced when PEG grafting density increased. Moreover, recent studies showed that human body is able to produce anti-PEG immunoglobulin (IgM); in particular, the production of anti-PEG IgM increased when the lipid dose increased [75]. However, studies on the recent vaccine for COVID-19 treatment demonstrated that the increase of anti-PEG IgM was recorded only after the first dose and no severe adverse reactions were observed in subjects that presented high levels of anti-PEG IgM even before the first dose administration [76]. Accelerated blood clearance (ABC) phenomenon is induced especially when repeated injections occur and in the future can affect the technique of PEGylation because of the increase of PEGylated products in the pharmaceutical field; indeed, the pre-existing anti-PEG IgM level has increased from 0.2 to 70% in the last 40 years [77]. PEG alternatives have been proposed, such the insertion of polyglycerol (PG), poly(oligo(ethylene glycol)methyl ether methacrylate) (POEGMA) and zwitterionic polymers [78]. Lila et al. [79] investigated the behaviour of PG-coated with respect to the ABC phenomenon. They produced both PEGylated liposomes (using mPEG_2000_-DSPE) and PG-liposomes (PG-DSPE) using the thin film hydration method, and encapsulated doxorubicin as model compound for cancer treatment. In vivo experiments highlighted that, after a first dose, the blood-circulating time was higher in the case of PEGylated liposomes with respect to PG-liposomes; but, after a second dose, the subjects showed a rapid clearance from the blood (4 h) and the uptake by the liver and spleen increased. Instead, subjects that received PG-coated liposomes as second dose did not show difference in blood concentrations, underlying that ABC phenomenon did not take place using PG-coated liposomes. Table 1 reports a summary of the drug encapsulated, kind of application, production technique and characteristics of liposomes discussed in this paragraph.

**Table 1:** Summary of the drug encapsulated, kind of application, production technique and characteristics of liposomes obtained in the different works described

LIPOSOMES						
Encapsulated drug	**Application**	**Production technique**	**Type of lipid**	**Size** **[nm]**	**ζ-potential** **[mV]**	**Ref.**
Antigenic peptide delivery	Cancer vaccines	Thin film hydration	DOTAP, DOPC	From 125 to 180	From + 20 to + 32	[67]
Ovalbumin	Vaccine	Thin film hydration	DOTAP, DSPE-PEG_2000_	278–299	From + 3 to + 36	[68]
Antigen	Cancer vaccines	Thin film hydration	HEPC, DSPE-PEG_2000_	110	-	[69]
Ovalbumin	Cancer immunotherapy or vaccination	Thin film hydration	PC, DOTAP	From 150 to 208	From − 9 to -50	[70]

### Transfersomes-based vaccines

**Fig. 4:**
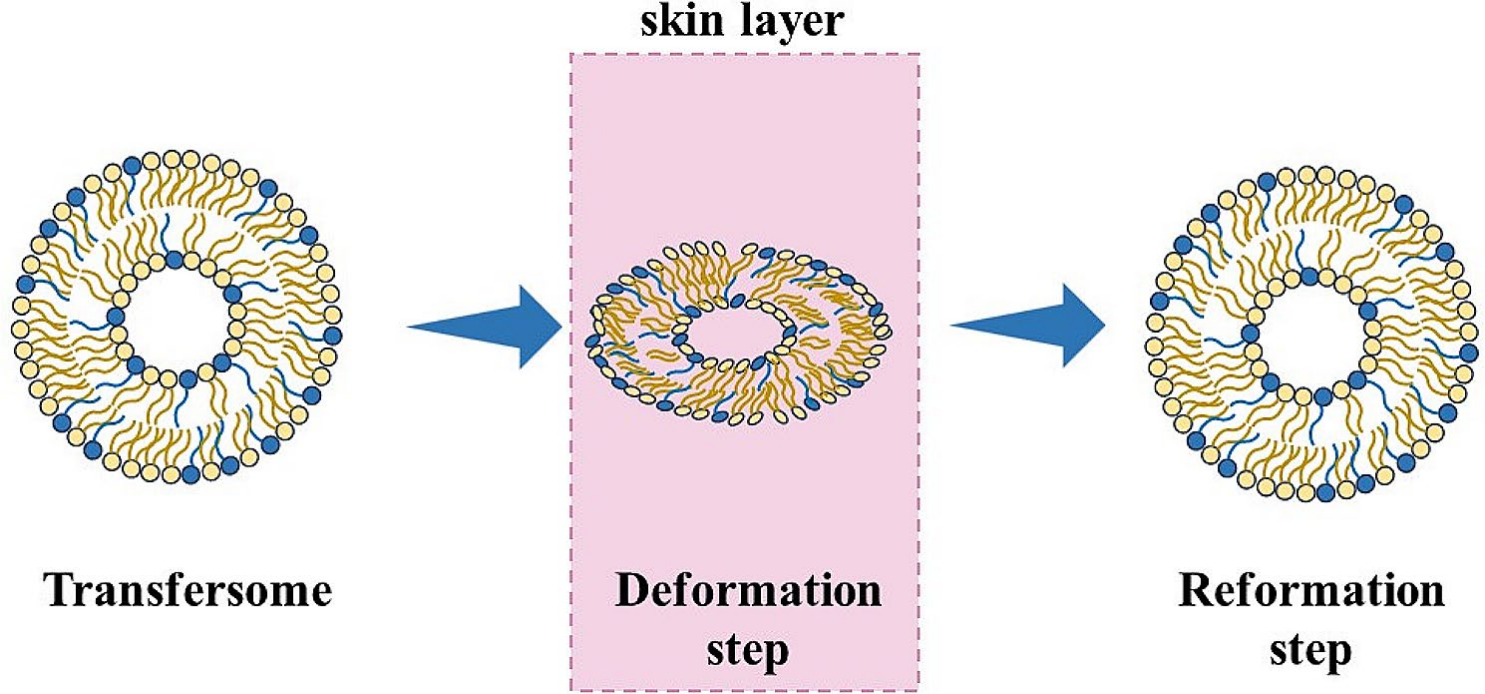
Mechanism of transfersomes permeation through the skin layer

Transdermal route is an easier way to deliver vaccines with respect to subcutaneous or intramuscular ones; moreover, transcutaneous immunization shows better immunogenicity [80] and has particular interest in using nanoparticles, with a diameter of about 100 nm, since they can move into hair follicles and deliver antibodies to APCs, like epidermal Langerhans cells. Particles-based vaccines for transdermal route can need a skin preparation to improve the permeability of the particles, such as: electroporation, sonophoresis, laser ablation and skin abrasion; but this can be a very aggressive and uncommon practice. To avoid the use of these invasive methods, transfersomes can be used for transdermal vaccine delivery; they are able to change their shape during the drug delivery and transportation throughout the skin (Fig. 4) [81]. The transport of transfersomes through the skin is due to the osmotic force or to the hydration. Mammalian skin can control hydration by generating a gradient between the epidermis and the dry stratum corneum; when the transfersome suspension is applied on the skin, a partial dehydration of vesicles occurs that makes them able to deform and pass through skin pores because of the hydration gradient: i.e., they penetrate in the more hydrated skin layer [82].

Gupta et al. [83] produced transfersomes formed by soya phosphatidylcholine (PC) as lipid and sodium deoxycholate as surfactant, using the thin film method, to be used for a non-invasive delivery system of tetanus toxoid. The obtained vesicle suspension was extruded through different polycarbonate membranes (dimension less than 0.45 μm) to reduce the vesicles dimension, although part of the suspension was inevitably lost. Different ratios of PC: surfactant were investigated to increase the deformability of the vesicles. In this work, elasticity of transfersomes was expressed throughout the deformability index, measured by extruding the vesicles through a 50 nm pore membrane and it was proportional to the amount of suspension extruded in a certain time (J), the radius of the transfersomes (r_v_) and the size of the membrane pore (r_p_) (Eq. 2):

2 $$ Deformability\,index\propto J\cdot {\left(\frac{{r}_{v}}{{r}_{p}}\right)}^{2}\left(2\right)$$

This is not the only way to define the deformability index; other definitions are widely used in the literature [84, 85]. Membranes are generally used since, to pass the mammalian skin, transfersomes must be capable to cross pores with a diameter less than 50 nm [86]. The results of this work showed that increasing the surfactant concentration over a limit value did not give advantages in terms of deformability index; the optimum ratio PC: surfactant was equal to 85:15. Fluorescence microscopy, using 6-carboxyfluorescein loaded transfersomes was performed using a rat skin and showed that the transfersomes penetrated even in a deeper skin layer. In vivo study demonstrated the effective immune response of the transfersomes that was comparable with the traditional intramuscular administration.

The efficacy of immune response can be affected by the kind of phospholipids used; as discussed before for liposomes, cation lipids were used to have an effective delivery of the antigen to the APCs. Also in this case, cation transfersomes are preferred, even for a better interaction with the negatively charged skin stratum corneum [26]. Mahor et al. [87] used cation lipid DOTMA (N-[1-(2,3-dioleyloxy)-propyl]-N, N,N-trimethylammonium chloride) and sodium deoxycholate as a surfactant, to produce vaccines against hepatitis B. Transfersomes were obtained by a modified reverse phase evaporation technique and the final suspension was extruded using a 100 nm polycarbonate filter. Subsequently, these transfersomes were introduced in a suspension containing plasmid DNA to permit the adsorption of negatively charged plasmid DNA on the transfersomes surface. These authors obtained nanovesicles characterized by an average diameter between 85 and 91 nm, and a drug encapsulation efficiency from 69% up to 88%.

However, the literature reports that the only application of transfersomes do not increase immunogenicity, and a skin preparation with microneedles is needed [80]. Microneedles are used to create a route for nanoparticles to penetrate the skin in a painless way [88]. In this regard, Wu et al. [89] prepared cationic and anionic transfersomes integrated with microneedles, encapsulating ovalbumin (OVA), as a model antigen. Egg yolk phosphatidylcholine was used as a lipid, and sodium cholate (SC) and stearylamine (SA) were used as surfactants, to obtain anionic and cationic transfersomes, respectively. They were produced using the thin film hydration method, and multilayer nanovesicles with dimensions ranging from about 122 nm to 199 nm, and a drug encapsulation efficiency of about 78%, were obtained in the case of cationic transfersomes. Microneedles were prepared using hyaluronic acid (HA) at different molecular weights in a micromold and dried at room temperature. To prepare transfersomes loaded microneedles, the HA powder was first dissolved in the nanovesicles suspension and, then, put in the mold and dried. Microneedle patches showed a complete dissolution after 20 min of application on rat skin, proving a high dissolving capacity in the skin. In contrast with the literature, in this case, a larger uptake from dendritic cells was reached by the anionic transfersomes; but it could be related to HA modification. Table 2 reports a summary of the drug encapsulated, kind of application, production technique and characteristics of transfersomes discussed in this paragraph.

**Table 2:** Summary of the drug encapsulated, kind of application, production technique and characteristics of transfersomes obtained in the different works described

TRANSFERSOMES						
Encapsulated drug	**Application**	**Production technique**	**Type of lipid/surfactant**	**Size** **[nm]**	**ζ-potential** **[mV]**	**Ref.**
Tetanus-toxoid	Topical immunization	Thin film hydration	PC, sodium deoxycholate	172–195	-	[83]
Plasmid DNA	Hepatitis B vaccines	Reverse phase evaporation	DOTMA, sodium deoxycholate	85–91	From − 1 to + 8	[87]
Ovalbumin	Evaluation of antigen productions via microneedles	Thin film hydration	PC, sodium cholate, steatrylamine	122–199	+ 40	[89]

### Niosomes-based vaccines

Niosomes represent another system for drug delivery and skin immunization; thanks to the surfactant characteristics, they are able to improve their fluidity throughout the stratum corneum [90]. They can be formed by the same surfactant or different surfactants and the most used ones are Span, Tween and Brij. Differently from liposomes, niosomes present a longer stability and are cheaper because of the use of surfactants [91].

The adjuvant activity of niosomes was studied for the first time by Brewer et al. [92], when they found the enhancing activity of niosomes in antibody production against bovine serum albumin (BSA); in particular, these authors found that niosomes were stimulators in type 1 T cells helper (Th1) response. Vyas et al. [93] presented a study where liposomes and niosomes encapsulating DNA encoding hepatitis B surface antigen (HbsAg) were produced and their immune actions were compared. Both liposomes and niosomes were obtained by the reverse phase evaporation method and were characterized by a mean size of 2.8 μm and 2.3 μm, respectively. The drug encapsulation was about 49% for liposomes and 45% for niosomes. The immune response was investigated, and niosomes showed a larger action than liposomes, with a larger production of specific antibodies (anti-HbsAg) in serum after topical application. Moreover, even intramuscular HbsAg was injected, showing a better response for the first 4 weeks; but, after that period, antibodies decreased in serum, whereas, in the case of liposomes and niosomes, the initial immune response was lower and increased after 8 weeks from the topical administration. Obeid et al. [94] showed that the immune response can be influenced by the nanocarrier preparation method. They produced niosomes using the thin film hydration method and microfluidic, to encapsulate influenza antigen. Thin film hydration method showed a drug entrapment efficiency of about 48%; whereas, it was about 57% in the case of niosomes produced via microfluidic. Moreover, larger nanovesicles were produced using the thin film hydration method (Z-average = 388.8 nm); whereas, a narrower size distribution and a smaller Z-average (122.1 nm) were obtained by microfluidic. The difference in size and encapsulation efficiency can affect the action of produced nanovesicles. Both vesicles did not show a significative difference with the free antigen in Th2 response, and this could be related to the low encapsulation efficiency obtained. Niosomes produced by the thin film hydration showed instead a larger Th1 response and this result was related to the mean dimension of the vesicles [92, 95]. In a recent work of Hassouna et al. [96], niosomes were prepared via thin film hydration method, using cholesterol and Span 60, and loaded with toxoplasma lysate antigen (TLA), to be used for the treatment of rheumatoid arthritis. Negatively charged nanovesicles (Z-average 164.12 nm) were obtained, and a 75% drug encapsulation efficiency was measured.

As discussed previously for transfersomes, the permeation properties of niosomes can be enhanced using microneedle patches that can be also auto-administrated [97]. Boonada et al. [98] prepared cationic niosomes to enhance skin immunization via hollow microneedle. Cationic niosomes formed by Span 20, cholesterol and a cationic lipid (N1, N1-dimyristeroyloxyethyl-spermine), in a molar ratio 2.5:2.5:0.5 mM, were produced using the thin film hydration method, and plasmid DNA-encoding ovalbumin was encapsulated. After sonication, particles were still of large dimensions because of the adsorption of anionic plasmid DNA on the cationic niosomes surface. In vivo studies were performed to determine the efficacy of the nanovesicles produced; high levels of IgG antibodies in all mice vaccinated with the microneedles were obtained. Moreover, no infections or bleeding were observed on the skin area where the patch was used. Zhang et al. [99] produced carbomer hydrogel niosomes for transcutaneous vaccine delivery. Niosomes (using Span 80, cholesterol and sterylamine) were produced by the thin film hydration method and were characterized by a mean diameter of about 300 nm and an ovalbumin encapsulation efficiency of about 49%. Then, the niosomal suspension was added to a solution of Carbopol 934 in PBS to obtain a loaded hydrogel, whereas the niosomal gel was obtained by adding niosomal suspensions to a solution of Carbopol in 20% ethanol/PBS. Hydrophobic and hydrophilic membranes were used to simulate the skin layer. The hydrophilic layer presented more resistance to the penetration of the niosomal gel; whereas, the niosomal hydrogel delivered antigens even into the deeper hydrophilic layer. Table 3 reports a summary of the drug encapsulated, kind of application, production technique and characteristics of niosomes discussed in this paragraph.

**Table 3:** Summary of the drug encapsulated, kind of application, production technique and characteristics of niosomes obtained in the different works described

NIOSOMES						
Encapsulated drug	**Application**	**Production technique**	**Type of surfactant**	**Size** **[nm]**	**ζ-potential** **[mV]**	**Ref.**
DNA	Hepatitis B vaccine	Reverse phase evaporation	Span 85	2300	-	[93]
Influenza antigen	Vaccine	Thin film hydration/microfluidic	Monopalmitin, dicetyl phosphate	388–122	From − 14 to -28	[94]
Toxoplasma lysate antigen	Inflammatory arthritis	Thin film hydration	Span 60	164	-57	[96]
Plasmid DNA-encoding ovalbumin	Skin immunization via microneedles	Thin film hydration	Span 20, N1, N1-dimyristeroyloxyethyl-spermine	400–600	-	[98]

### A comparative study for non-invasive vaccine delivery

A comparative study to better understand which non-invasive delivery strategy could be the best one among liposomes, transfersomes and niosomes, was proposed by Gupta et al. [100]. Liposomes and niosomes were produced by the reverse phase evaporation method; whereas transfersomes were produced by the thin film evaporation method. In all the cases, the obtained suspension was extruded throughout polycarbonate filters of different porosities to control the dimensions of the nanovesicles; the size measured for transfersomes, liposomes and niosomes was 196 nm, 221 nm and 214 nm, respectively. Encapsulation efficiency and deformability were higher only for transfersomes (72% drug encapsulation of transfersomes against 42% and 41% for liposomes and niosomes, respectively, and a deformability index of one order of magnitude higher than the one calculated for liposomes and niosomes). In vivo results highlighted the superior action of transfersomes in the case of topical administration with respect to niosomes and liposomes; indeed, transfersomes exhibited an immune response similar to that of intramuscular administration of naked antigen, whereas niosomes and liposomes showed a weaker immune response. The fact that, in this study, transfersomes did not give a larger immune response than the injection administration can be related to the method of preparation, as well as the weaker action of niosomes can be related to the low encapsulation efficiency of the antigen. For these reasons, transfersomes can be considered the best choice for transdermal delivery: they can enhance the permeation of both low and high molecular weight compounds [101]; however, also niosomes, as reported before, could be used for transdermal delivery, but more studies and optimized formulations are needed to improve their elasticity. Liposomes do not present deformable properties because of their nonelastic features [102]; therefore, they can be preferred in the case of intramuscular administration. Nevertheless, further studies have to be performed and compared to verify and validate this conclusion.

## Conclusions and perspectives

Liposomes, transfersomes and niosomes used to deliver mRNA and DNA-based vaccines show larger efficacy and reduced side effects than the traditional vaccine formulations. In particular, transfersomes can be the best choice for transdermal delivery because of their elasticity and deformability properties.

The most used traditional methods for nanovesicles production are the thin film hydration method and the reverse phase evaporation; but some limitations occur due to their intrinsic batch-mode configuration, post-processing steps of sonication or extrusion to reduce the vesicle size, and purification procedures to remove organic solvent residues.

Supercritical CO_2_-based processes can overcome some of these problems. In particular, SuperSomes is a continuous and one-shot technique that was proposed to produce liposomes, transfersomes and niosomes encapsulating both hydrophilic and lipophilic drugs. Adopting this approach, the industrial requirements can more readily be satisfied; but, further studies have to be performed to validate the application of this process to the treatment of high added value molecules, like RNA and DNA.

## Data Availability

Not applicable.

## Abbreviations

ABC: Accelerated blood clearance
Ag: Antigen
APC: Antigen presenting cells
CPP: Critical packing parameter
DOPC: 1,2-dioleoyl-sn-glycero-3-phosphocholine
DOTAP: 1,2-dioleoyl-3-trimethylammonium-propane
HA: Hyaluronic acid
HbsAg: Hepatitis B surface antigen
HEPC: Hydrogenated egg phosphatidylcholine
Ig: Immunoglobulin
OVA: Ovalbumin
PC: Phosphatidylcholine
PEG: Polyethylene glycol
PG: Polyglycerol
POEGMA: Poly(oligo(ethylene glycol)methyl ether methacrylate)
SA: Sterylamine
SC: Sodium cholate
Th1: Type 1 T helper
Th2: Type 2 T helper
